# Epidemiological profile of perinatal mental disorders at a tertiary hospital in Yaoundé- Cameroon

**DOI:** 10.3389/fgwh.2023.999840

**Published:** 2023-02-01

**Authors:** Joël Djatche Miafo, Namanou Ines Emma Woks, Daniel Nzebou, Idriss Tchaptchet, Suzi Thio Delene, Orelien Kegha Tchidje, Gervais Ndzodo, Berthe Siewe Kamga, Lucienne Bella Assumpta

**Affiliations:** ^1^ Research Department, Uni-Psy et Bien-Être (UNIPSY), Yaoundé, Cameroon; ^2^ Yaoundé Gynaecology, Obstetrics and Pediatrics Hospital, Yaoundé, Cameroon

**Keywords:** maternal health, maternal mental health, perinatal mental disorders, risk factors, Cameroon

## Abstract

In developing countries, 15.6% of pregnant women and 19.8% after childbirth experience a mental disorder. In the absence of data on the situation in Cameroon, we carried out a study to determine the prevalence of perinatal mental illness in this hospital and its risk factors among women in perinatal period and the relationship between both at the Yaoundé Gynaeco-Obstetric and Paediatric Hospital, a reference mother and child hospital. We conducted a hospital-based, cross sectional, observational study. Data was collected using structured and semi-structured interviews. There were six sub-themes covered: participants’ socio-demographic profile, clinical profile, perinatal history, psychopathology aspects with the Mini International Psychiatric Interview, the Edinburgh Postnatal Depression Scale, the State Trait Anxiety Inventory and the perinatal mental illness risk factors. Data entry was done using Microsoft Excel 2010 and transferred to Statistical Package for the Social Sciences version 23.0 for analysis. Among 194 women who participated in the study, the general prevalence for perinatal mental disorders was 53.6% (104/194), 25.8% among pregnant women and 27.8% among postnatal women. Comorbidities were present in 17.5% of our study population. We observed that 45.8% suffered from depression, 17% had a risk of suicide, 10.3% suffered from perinatal anxiety, 3.1% presented with post-traumatic stress disorder, 3.6% acute stress disorder, 7.7% had adjustment disorder. Concerning risk factors, we found a significant link between depression and severe anxiety before delivery (*p* < 0.05) and the absence of social support (*p* = 0.005). We found that women with at least four risk factors were 1.6 times more likely to present with a perinatal mental disorder. The prevalence of perinatal mental disorders at this Hospital is very high. This highlights the need for institutional screening and management of perinatal mental disorders, which suggests that we explore the situation in others and other health facilities in Cameroon.

## 1. Introduction

The perinatal period starts at 22 completed weeks of gestation and ends seven completed days after birth as described by the World Health Organization (WHO) (1). When broadly defined, this period extends from conception to 12 months after childbirth (2). Some women develop psychiatric disorders during pregnancy, while others do in the post-partum period. This group of disorders is known as perinatal mental illnesses (3). It ranges from depression and anxiety disorders during pregnancy to maternity blues, post-partum depression, bipolar disorder and post-partum psychosis during the puerperal period (4). About 10% of pregnant women and 13% of women who have just given birth experience a mental disorder worldwide. In developing countries, this is even higher: 15.6% during pregnancy and 19.8% after childbirth (5, 6). According to the WHO, one in five women suffer from this in developing countries (6). In Cameroon, two studies conducted at the Yaoundé Gynaeco-Obstetric and Paediatric Hospital (YGOPH) in Cameroon (7, 8)- whose objective was to screen for postpartum depression and postpartum blues. They reported a prevalence of 23.4% and 33.3% respectively. Further, a study of teenage mothers in Cameroon revealed that, 70% of 1,365 teenage mothers, had score of 13 or higher, in the Edinburgh Postpartum Depression Scale (9). These perinatal mental disorders in Cameroon are generally associated with risk factors such as: unintended pregnancy, young age, being unmarried, lacking intimate partner support, having hostile in-laws, poor socioeconomic status, experiencing domestic violence, having a history of mental health problems and many others (10–12). Indeed, the potential negative impact on maternal and child outcomes makes the perinatal period a critical time to identify psychiatric illnesses (13–18). However, there is still a paucity of data concerning the general burden of perinatal mental disorders at the YGOPH (19, 20). It is important to identify these conditions early and treat to avoid poor maternal, foetal and infant outcomes, but there is still limited data published on the clinical assessment of women in the pre- and postnatal period for mental disorders. Therefore, to fill this gap and to improve the mental health of pregnant and breastfeeding women in Cameroon, we decided to conduct this study at the YGOPH in Cameroon, which receives a huge influx of women. The general objective of our study is to determine the epidemiological profile of perinatal mental disorders at YGOPH. its aims are to describe the socio-demographic characteristics of participants, to determine the prevalence of perinatal mental illness, to identify the risk factors of mental disorders among women in perinatal period who deliver at YGOPH, and the relation-ship between both.

## 2. Methods

### 2.1. Ethical approval

The study proposal was approved by the Institutional Ethics and Research Committee for Human Health (CIERSH), and the approval number was 1021/CIERSH/DM/2020. All participants signed an informed consent form prior to participating in the study and were informed that they could withdraw from the study at any time. Confidentiality was strictly respected. Study procedures were implemented in conformity with ethical principles as set out in the Declaration of Helsinki (21).

### 2.2. Study design and period

We conducted a hospital-based, cross-sectional study. It was a collaboration between YGOPH and the UNIPSY organization (Uni-Psy et Bien-Être), a group of mental health professionals in Cameroon. The study was conducted over a period of 1 month, from 13 April 2020 to 13 May 2020.

### 2.3. Study setting

The study was carried out at the YGOPH. It is a tertiary mother and child hospital located in the Centre region of Cameroon. It is the biggest hospital dedicated to maternal and pediatric care in the region. It has a gynaecology and obstetrics service organized as follows: an outpatient gynaecology unit where about 3,000 women are seen at antenatal consultations monthly and, a maternity and gynaecology admission unit which records approximately 300 deliveries per month. The hospital also has a paediatrics service with an outpatient unit, two admission units and a vaccination unit.

### 2.4. Participants

Our study population comprised all pregnant women who consulted at the YGOPH during the study period and women from 0 to 12 months’ post-partum. Our sample was made up of patients who either gave birth or attended antenatal consultation (ANC) and women who returned with their babies for vaccination or paediatric consultation during the study period. Indeed, either they were referred to us by doctors or nurses, or they volunteered themselves after an awareness campaign that we did in the waiting room. The inclusion criteria were: be a female patient consulting at YGOPH, be a pregnant woman irrespective of her gravidity or parity, be a woman from zero to 12 months’ post-partum regardless of the outcome of the pregnancy and women who gave their signed consent. The exclusion criteria were patients who did not consent to participate in the study.

#### 2.4.1. Sampling

After calculating the minimum sample size, participants were recruited into two different sub-groups. In one sub-group, we had pregnant women attending our health facility for antenatal care and in the other, post-partum women hospitalized following delivery or visiting YGOPH for vaccination or paediatric care during the first 12 months. The minimum sample size to be attained was shared proportionately between the two groups. Once the target number of participants was attained for antepartum and postpartum women, recruitment stopped. To calculate the sample size, we used the Lorentz Formula: *N* = *p* (1-*p*) (*zα*/*d*)^2^, *p*: prevalence of antenatal psychiatric disorders among women attending antenatal clinic at a tertiary maternity hospital in Ilorin, Nigeria over a period of 8 weeks (19). *p* = 12.5%; *d*: degree of precision: *d* = 0.05; *Zα*: normal distribution value: *Zα *= 1.96; *N*: sample size. *N* = 0.125 (1–0.125) (1.96/0.05)^2^ = 168; adjusted minimum sample size: *N*’ = (*N*)/(1-q), where, *N*’ is the adjusted/corrected minimum sample size, *N* is the initial minimum sample size, q is the probable proportion of a poorly filled questionnaire (10%). We worked with an adjusted minimum sample size of 190 cases: 95 pregnant women and 95 postpartum women.

### 2.5. Procedure

The research proposal was drafted, corrected and submitted to obtain ethical clearance. An online discussion forum was created for the research project to facilitate information exchange and coordination of the project. A 3-day training was organized with the data collection team (five psychologists), educating them on how and when to conduct the interview, including the questionnaire and interview guide. Data collection was done 3 days a week until the quota was attained for each subgroup. Data was collected using both structured and semi-structured interviews under five sub-themes as presented (Table 1):

**Table 1:** Sub-themes used for data collection.

Sub-themes	Contents
Socio-demographic profile	Age, level of education, marital status, profession, type of family
Biomedical profile	gravidity, parity, gestational age, illness…
Perinatal history	context with the spouse, the state of their relationship and social interactions with others during the perinatal period, of the pre-, per- and post-partum experience, the changes and transformations related to parenting, and the quality of the mother-baby relationship
Psychopathology aspects and mental disorders	Tools of screenning: Mini International Psychiatric Interview (MINI), the Edinburgh Postnatal Depression Scale (EPDS) and Spielberger State Anxiety Questionnaire (STAI)
Perinatal mental illness risk factors	Five catgories: circumstances of pregnancy and maternity, health issues, the social risk factors, reproductive/childbirth experience, the marital and family relationship category

### 2.6. Study tools

These tools were chosen according to the objectives of the study and their scientific and clinical relevance.

#### 2.6.1. Spielberger state anxiety questionnaire (STAI)

The State-Trait Inventory for Cognitive and Somatic Anxiety was designed to assess cognitive and somatic symptoms of anxiety as they pertain to one's mood in the moment (state) and in general (trait) (22). This scale was used to assess the anxiety level of women, with the objective of screening for perinatal anxiety. It was self-administered by all, except those who could not read or write. In the latter case, the statements were read out to each participant, and those which corresponded to the participant's feelings were marked with a cross in one of the four columns on the right. Concerning the scoring system used on the rows labelled I, the answer “no” was rated 4, “more or less no” was rated 3, “more or less yes” was rated 2 and “yes” was rated 1. For the rows labelled 0, the “no” was rated 1, “more or less no” = 2, “more or less yes” = 3, “yes” = 4. The total score therefore varied from 20 to 80. The interpretation was as follows: below 35: anxiety level was minimal: calm nature. From 36 to 45: anxiety level was low. From 46 to 55: level of anxiety was moderate. From 56 to 65: the level of anxiety level was high, significant and the intervention of a professional required. Above 66: the anxiety level was very high. In our study, we considered the score 56 and above as a state reflecting significant anxiety in mothers.

#### 2.6.2. Edinburgh postnatal depression scale (EPDS)

This scale is mainly used to screen for perinatal depression (23, 24). Originally, it was developed to screen postnatal depression. Otherwise, it is also used to screen antenatal depression (24). It's a 10-item self-report questionnaire in which each question is scored 0–3 (resulting range 0–30). Items 1, 2, & 4 (without an *) are scored 0, 1, 2 or 3 with top answer proposal as 0 and the bottom answer proposal scored as 3. Items 3, 5–10 (marked with an *) are reverse scored, with the top answer proposal scored as 3 and the bottom answer proposal scored as 0. A cut-off at 12/13 is usually suggested in the literature for “possible depression” (25). A cut-off at 12 was chosen in this study (24).

#### 2.6.3. Mini international psychiatric interview (MINI)

The Mini-international neuropsychiatric interview (MINI) is a short structured clinical interview which enables researchers to make the diagnoses of psychiatric disorders according to DSM-IV (26). The duration of the interview was approximately 15 min and was designed for epidemiological studies and multicentre clinical trials (27). The MINI assesses 14 mental health problems. It also makes it possible to make a diagnosis when used properly. It measures the absence or presence of disease, often based on stringent criteria that meet those in the Diagnostic and Statistical Manual of Mental Disorders (DSM) (28, 29). In this study, we used the French version 5.0.0 whole life.

#### 2.6.4. Psychodynamic diagnostic manual (PDM)

The Psychodynamic Diagnostic Manual (PDM) is a diagnostic handbook similar to the International Classification of Diseases (ICD) (30) or the Diagnostic and Statistical Manual of mental disorders (DSM) (27). The PDM aims to detect and describe patients’ characteristic mental experiences. It attempts to restore the connection between deep or psychodynamic understanding and treatment, without the requirements of other diagnostic systems that they be useful for demographic studies, billing, institutional record-keeping, syndromes research, and other ancillary uses of diagnostic labels (28, 31). We used it particularly in this study to diagnose other psychological pathologies not included in the MINI, especially maternal affective disorders such as: perinatal depression, puerperal psychosis and the subjective experience of maternal affective disorders (affective states, cognitive patterns, somatic states, relationship patterns).

In brief, we relied on the MINI test to diagnose mental disorders and suicide risk. More specifically, diagnoses of acute stress disorder and adjustment disorder were made using the PDM. For perinatal depression, we used 2 tools: the EPDS and the PMD. Indeed, a score of at least 13 on the EPDS and a positive diagnosis on the PDM further strengthened the diagnosis of perinatal depression, even if the PMD alone was sufficient. The diagnosis of perinatal anxiety was based on a score of at least 56 on the STAI.

#### 2.6.5. Risk factors of perinatal mental disorders inventory

A risk factor is any attribute, characteristic or exposure of a subject that increases the likelihood of developing an illness or suffering a trauma. An inventory of 24 risk factors was constructed and categorized. We considered five categories of risk factors documented in other studies (10, 11, 32). These categories are: circumstances of pregnancy and maternity with 6 factors (teenage pregnancies, unwanted or unplanned pregnancies, pregnancy as a result of rape, being single or separated, large number of children, birth of a girl in cultures preferring a boy child). then we have the category of health issues with 4 items, the social risk factors with 4 items, the category reproductive/childbirth experience with 4 factors. And finally, the marital and family relationship category containing 6 factors (see Table 3). We administered this inventory to all mothers in our sample and measured the number of mothers observed for each category and the proportion of each factor compared to all the factors in the category.

### 2.7. Data analysis

Data entry was done using Microsoft Excel 2010 and transferred to SPSS version 23.0 for analysis. The analysis consisted of highlighting the descriptive statistics (frequency and percentage) of study variables. We performed a logistic regression between having pathology or not, and risk factors during the perinatal period. All risk factor variables were introduced by the top-down stepwise method. The aim was to find out which factors can predict the occurrence of mental pathologies. Then, we evaluated the risks (Odds Ratio) between the dichotomous variables; and performed the Chi square test (*X*^2^) to test the association between the socio-demographic data and the clinical characteristics. The result was statistically significant when its value was less than 0.05.

## 3. Results

### 3.1. Sociodemographic and clinical characteristics of participants

For a total of 194 women who participated in the study, the proportion of women in the antenatal and postnatal groups were 94 (48.5%) and 100 (51.5%) respectively. The most represented age group was 21–30 years. The majority of these women were married (37.6%). Regarding their level of education, the proportion of women with a university degree (52.1%) was highest. Similarly, more than 50% of participants were employed, while the rest were unemployed. Most of them were multigravida (70.6%), and 47% were pregnant and 53% had already given birth (Table 3).

### 3.2. Perinatal mental disorders

**Figure 1:**
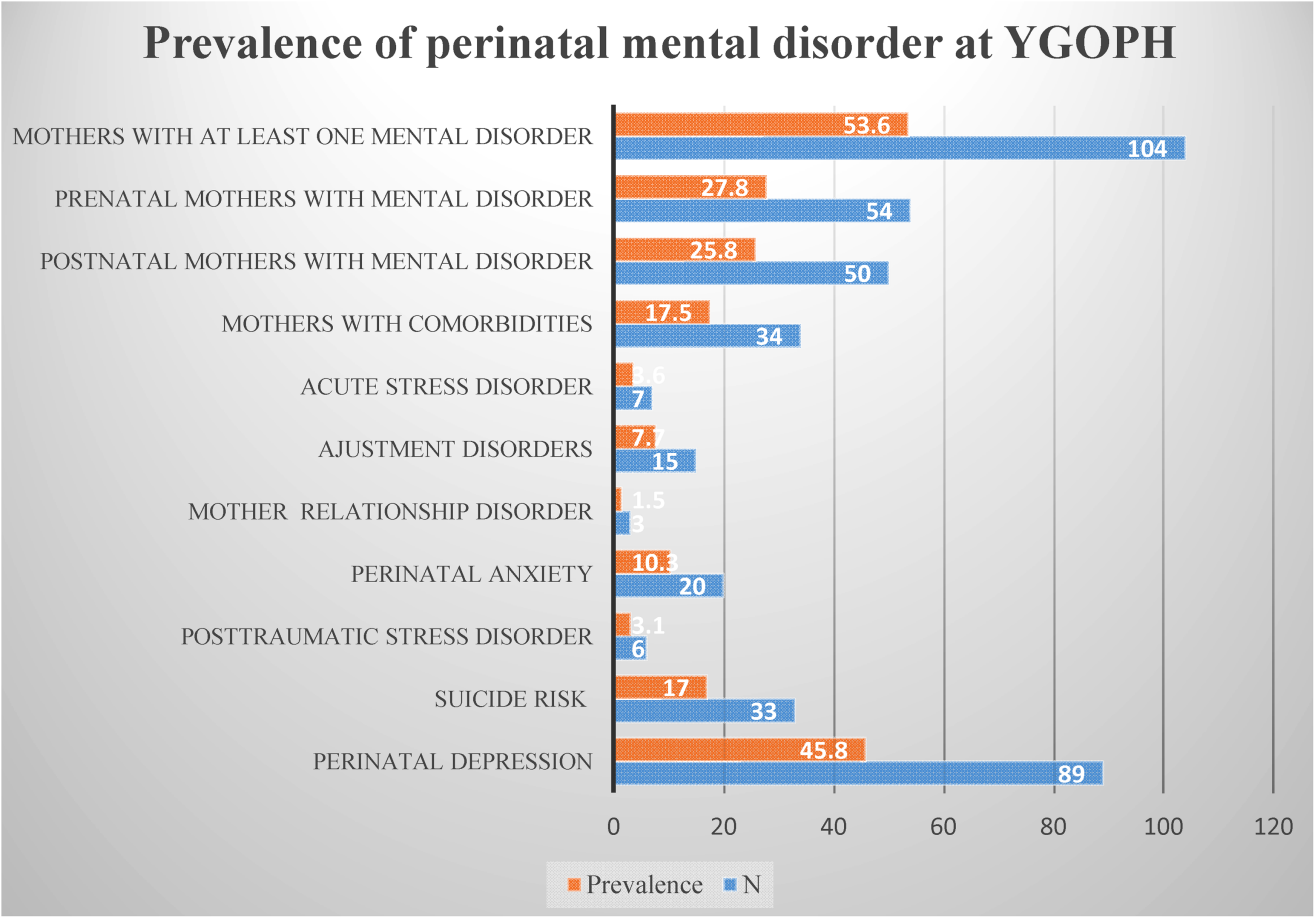
Instantaneous prevalence of perinatal mental disorders at YGOPH.

The prevalence of perinatal mental disorders in our study population was 53.6% (*N* = 104). More precisely the prevalence was 53.1% (*N* = 50) in prenatal mothers and 54% (*N* = 54) in postnatal mothers. The proportion of comorbidities was 17.5%, distributed as follows: 12.9% had two psychological disorders and 4.6% had three. For most of these associated disorders, perinatal depression and perinatal anxiety or one of them was associated with another mental disorder. Concerning the prevalence of each psychological disorder in the total study population, we observed that 45.8% suffered from depression respectively 43.6% (*N* = 41) of mothers prenatally and 48% (*N* = 48) postnatally. 10.3% experienced perinatal anxiety, including 10.6% (*N* = 10) in prenatal and 10% (*N* = 10) in postanal. 3.1% (*N* = 6) mothers presented with post-traumatic stress disorder, i.e., 5.3% (*N* = 5) in antenatal and 1% (*N* = 1) in postnatal. 3.6% (*N* = 7) with acute stress disorder diagnosed, all in pregnant. Fifteen women had adjustment disorder, including 7.4% (*N* = 7) in pregnancy and 8% (*N* = 8) in post-partum. Finally 1.7% (*N* = 3) with dysfunctional mother-child (ren) relationships, all in postpartum. Excluding these pathologies diagnosed, we note that 17% (*N* = 33) had a risk of suicide (Figure 1).

### 3.3. Risk factors of perinatal mental disorders

The frequency of risk factors and the logistic regression predicting perinatal psychological disorders are presented in Tables 2, 4.

**Table 2:** Risk factors of perinatal mental disorders at the YGOPH.

Associated factors (variable and category)	1) Prenatal		2) Postnatal		Total
	Frequency	Study population *N* (%)	Frequency	Study population *N* (%)	*N* (%)
***Context of motherhood (N = 111*)**	53	47.74	58	52.25	
Teenage pregnancies	5	4.50	4	3.60	9 (8.1)
Unwanted pregnancies	26	23.42	31	27.92	57 (51.4)
Pregnancy as a result of rape	1	0.90	1	0.90	2 (1.8)
Being single or divorced	14	12.61	18	16.21	32 (28.8)
Many children	3	2.70	2	1.80	5 (4.5)
Birth of a girl instead of a boy	4	3.60	2	1.80	6 (5.4)
***Medical history (N = 174)***	79	45.40	95	54.59	
History of mental illness	24	13.79	17	9.77	41 (23.5)
Depression before delivery	29	16.66	48	27.58	77 (44.3)
Illness during pregnancy	21	12.06	22	12.64	43 (24.7)
HIV positive	5	2.87	8	4.59	13 (7.5)
***Social factors (N = 244)***	112	45.90	132	54.09	
Lack of social support	14	5.73	20	8.19	34 (13.9)
Poverty	18	7.37	18	7.37	36 (14.8)
Refugees/displaced/migrants	1	0.40	2	0.81	3 (1.2)
War zone, conflict, natural disasters (COVID-19)	79	32.37	92	37.70	171 (70.1)
***Reproductive experience (N = 126)***	57	45.23	69	54.76	
History of miscarriage	32	25.39	27	21.42	59 (46.8)
Recurrent miscarriages	5	3.96	5	3.96	10 (7.9)
Sick babies	1	0.79	8	6.34	9 (7.1)
Pregnancy termination	19	15.07	29	23.01	48 (38.1)
***Marital/family relationship (N = 217)***	100	46.08	117	53.91	
Unsupportive husband	21	9.67	23	10.59	44 (20.3)
Polygamous family	10	4.60	7	3.22	17 (7.8)
Physical abuse	18	8.29	19	8.75	37 (17.1)
Inability to confide in partner	18	8.29	28	12.90	46 (21.2)
Difficult relationship with in-laws	23	10.59	28	12.90	51 (23.5)
Difficult relationship with family	10	4.60	12	5.52	22 (10.1)
***Number of risk factors (N = 194)***	94	48.45	100	51.54	
Mean = 4.51					
1) 1–3	41	21.13	39	20.10	80 (41.2)
2) 4–6	33	17.01	38	19.58	71 (36.6)
3) 7–9	17	8.76	18	9.27	35 (18.0)
4) >9	3	1.54	5	2.57	8 (4.1)

**Table 3:** Socio-demographic and clinical characteristics of participants.

Variable and category	1) Prenatal		2) Postnatal		Total	
	Frequency	Study population *n* (%)	Frequency	Study population *n* (%)	Frequency	Study population *n* (%)
**Sociodemographic**	90	47.61	99	52.38		
**Age (*N* = 189)**						
Mean = 29.36						
1) <21	5	5.55	5	5.05	10	5.29
2) 21–30	47	52.22	53	53.53	100	52.91
3) 31–40	38	42.22	38	38.38	76	40.21
4) >40			3	3.03	3	1.58
**Marital status (*N* = 194)**	94	48.45	100	51.54		
1) Single	21	22.34	38	38	59	30.4
2) Married	40	42.55	33	33	73	37.6
3) Divorced	1	1.06			1	0.5
5) Concubinage	19	20.21	22	22	41	21.1
6) Engaged	13	13.82	7	7	20	10.3
**Level of education (*N* = 194)**	94	48.45	100	51.54		
1) Primary school	5	5.31	6	6	11	5.6
2) Secondary school	45	47.87	37	37	82	42.3
3) University	44	46.80	57	57	101	52.1
**Profession (*N* = 194)**	94	48.45	100	51.54		
1) working	52	55.31	48	48	100	51.5
2) Not working	42	44.68	52	52	94	48.5
**Residence (*N* = 194)**	94	48.45	100	51.54		
1) Rural	5	5.31	4	4	9	4.6
2) Urban	89	94.68	96	96	185	95.4
**Type of family (*N* = 193)**	93	48.18	100	51.81		
1) Single parent	17	18.27	29	29	46	23.8
2) Monogamy	64	68.81	56	56	120	61.9
3) Polygamy	12	12.90	15	15	27	13.9
**Clinical**						
**Perinatal state (*N* = 194)**						
1) Prenatal					94	48.5
2) Postnatal					100	51.5
**Gravidity (*N* = 194)**	94	48.45	100	51.54		
1) Primigravida	25	26.59	32	32	57	29.4
2) Multigravida	69	73.40	68	68	137	70.6
**Parity (*N* = 193)**	93	48.18	100	51.81		
1) Nulliparous	14	15.05	3	3	17	8.8
2) Primiparous	23	24.73	45	45	68	35.2
3) Multiparous	49	52.68	43	43	92	47.6
4) Grand multiparous	7	7.52	9	9	16	8.2
**Gestational age (*N* = 153)**	94	61.43	59	38.56		
1) First trimester	10	10.63			10	5.2
2) Second trimester	25	26.59	1	1.69	26	13.4
3) Third trimester	45	47.87			45	23.2
4) Term pregnancy	10	10.63			10	5.2
5) Postdate	4	4.25	50	84.74	54	27.8
6) Post term			8	13.55	8	4.1
**Physical illness during pregnancy (*N* = 183)**	94	51.36	89	48.63		
1) Yes	34	36.17	41	46.06	75	40.98
2) No	60	63.82	48	53.93	108	59.01

**Table 4:** Link between risk factors and perinatal mental disorders at the YGOPH.

	A pathology (*n* = 104)	No pathology (*n* = 90)	aOR (CI 95%)	*p-value*
	*N* (%)	*N* (%)		
***Context of motherhood***				
Teenage pregnancies (*n* = 9)	8 (7.69)	1 (1.11)	7.417 (0.909–60.491)	*0*.*093*
***Health issues***				
Depression or severe anxiety before delivery (*n* = 77)	55 (52.88)	22 (24.44)	3.469 (1.874–6.423)	*0*.*000*
***Social risk factors***				
Lack of social support (*n* = 34)	26 (25)	8 (8.88)	3.417 (1.459–8.001)	*0*.*005*
***Reproductive experience***				
History of miscarriage (*n* = 59)	25 (24.03)	34 (37.77)	0.528 (0.284–0.981)	*0*.*092*
***Marital/family relationship***				
Inability to confide in partner (*n* = 46)	35 (33.65)	11 (12.22)	3.643 (1.720–7.716)	*0*.*087*
Difficult relationship with in-laws (*n* = 51)	35 (33.65)	16 (17.77)	2.346 (1.193–4.614)	*0*.*098*

The risk factors for perinatal mental disorders with the highest frequency were social risk factors with the highest sub-factors being residence in areas affected by war or conflict or natural disasters like COVID-19 pandemic (70.1%). Marital and family relationships were the next most occurring risk factor with a total of 217 observations and its most recurrent sub-factor was a difficult relationship with the mother-in-law (23.5%). Health issues followed closely with a total of 174 observations and the most recurrent sub-factor being depression or severe anxiety before delivery (44.3%). Negative experience during childbirth was next with a total of 126 observations and its most frequent sub-factor being a history of miscarriage or stillbirth (46.8%). Finally, a difficult context of pregnancy/motherhood was observed 111 times and the most present sub-factor was unwanted or unplanned pregnancies (51.4%).

One of our research objectives was to identify risk factors for perinatal mental disorders reported in other studies (32). We noted that these factors were common in our study population. The following categories of risk factors were present: social risk factors (*N* = 244), context of motherhood (*N* = 111), health issues (*N* = 174), negative reproductive experiences (*N* = 126) and marital and family relationships (*N* = 217). We performed a multiple logistic regression between the suspected risk factors and the presence or absence of a mental pathology. We found association with two factors: depression/severe anxiety before delivery (*p = 0.000; wald = 14.739; df = 1*) and the absence of social support (*p = 0.005; wald = 7.862; df = 1*). Moreover, mothers with these two risk factors had a three-fold greater risk of developing a perinatal psychological disorder [OR: 3.469 (1.874–6.423 CI 95%) and OR: 3.417 (1.459–8.001) CI 95%)]. Similarly, we found that women with at least four risk factors had a 1.6 higher likelihood of developing a mental pathology (OR: 1.664). This supports our results. In our research a history of mental illness and lack of practical support were the only risk factors significantly related to perinatal mental disorders. In the three studies conducted in Cameroon on the risk factors of perinatal depression and maternity blues (7–9) in adult and adolescent mothers, none of them identified an association between perinatal mental disorders and the absence of social support. Nonetheless, the two risk factors identified in our study were also reported in a meta-analysis on perinatal mental disorders and associated factors in low and middle-income countries (10). On the other hand, different risk factors mentioned in this meta-analysis (10), such as unfavourable socio-economic conditions, unplanned or unwanted pregnancies, single status were not associated with perinatal mental disorders in our study. Further, being pregnant and having a physical illness during pregnancy increased the chance of having a psychological pathology by 1.2 times (OR: 1.278; OR: 1.299). An association between gestational age and perinatal mental disorders was also observed (*p = 0.022*).

The above information gives us an overview of the frequency of perinatal mental disorders and the associated factors in a first category hospital in Cameroon and Central Africa. One of our research objectives was to obtain data on perinatal mental health, in order to address the problem of paucity of data on mental health in Cameroon and Central Africa in general. This information from our findings showing the hospital prevalence of perinatal mental disorders is new in Cameroon, because it was previously not available.

Overall, prepartum depression (*p* = 0.000) significantly increased a woman's having at least one mental illness during pregnancy. Similarly, lack of practical support (*p* = 0.005) also significantly increases the likelihood of having at least one mental illness for a woman during pregnancy. On the other hand, there was no statistically significant difference (*p* = 0.350; wald = 0.874; *df* = 1) between social risk factors and having at least one mental illness during pregnancy.

We also wanted to know from what number of risk factors the risk of mental pathology in mothers during the perinatal period could be raised. The intention was to know from what moment onwards we could monitor mothers more closely, or take preventive action. The results are presented in Table 5. It appears that with 4 or more risk factors, mothers have a 1.66 times greater chance of presenting a mental pathology. And with less than 4 risk factors, the relative risk is 0.49. In sum, mothers with risk factors have 0.29 times the chance of having mental illness.

**Table 5:** Odds ratio between risk factors and having a pathology during the perinatal period.

	A pathology (*n* = 104)	No pathology (*n* = 90)	*OR/RR*
***Mothers with risk factors (RF)***	104 (100)	90 (100)	0.296
Rf ≤ 3 (*n*: 80)	29 (36.2)	51 (63.7)	0.492
Rf > 3 (*n*: 114)	75 (65.7)	39 (34.2)	1.664

## 4. Discussion

Mental disorders are of great clinical significance among mothers in the perinatal period. The high prevalence (53.6%) of these disorders amongst women consulting at the YGOPH raises some concerns. This is higher than the overall average of 35.5% observed in developing countries (10). This is also higher than WHO's statistics (6, 10) which indicate that one out of five mothers in developing countries suffer from perinatal mental disorders. Moreover, while 10% of pregnant women and 13% of breastfeeding women suffer from mental disorders worldwide, a meta-analysis reports higher prevalence of 15.6% and 19.8%, respectively in developing countries (4, 10). Our findings (27.8% in the prenatal period and 25.8% in the postnatal period) also show higher prevalence of perinatal mental disorders when compared to the global figures.

Two studies on maternal mental health at YGOPH were found in literature (7, 8). Compared to these two studies, the novelty of ours is the inclusion of pregnant women and mothers beyond 6 weeks post-partum. Previous studies assessed only mothers seen during the first 6 weeks post-partum. In addition, we used professional screening (EPDS and STAI) and diagnostic tools (MINI and PDM) to assess other perinatal mental health conditions, whereas the former only used the EPDS to screen for postpartum depression. Regarding women with multiple of perinatal mental disorders (comorbidities), our results show that the prevalence is 17.5%. These statistics are different and slightly higher than the results of a South-African study (25), which found comorbidities in 11.9% of cases.

Considering the perinatal mental pathologies independently, our results show that perinatal depression had a high prevalence of 45.8%, and was the highest of all psychological pathologies. This is supported by the fact that 49% of the mothers interviewed had an EPDS score above 12. In the previous study conducted at YGOPH, the prevalence of post-partum depression symptoms was 23.4% (7), while in our study, it is 24.7%. Our results concerning the prevalence of perinatal depression at 45.8% are comparable to a study in Rwanda (33), which found a prevalence of perinatal depression at 50.3%.

Concerning suicidality, we also found that 17% of the mothers were at risk of suicide; 10.8% at low risk, 2.6% at medium risk and 3.6% at high risk. Moreover, we observed that it was three times (OR = 3.441) more likely for mothers with a sign of suicidality to present with a psychological pathology. This association between mental disorders and suicidal behaviours (suicidal ideation, wanting to harm oneself, suicidal plans and attempts) was found to be statistically significant (*p = 0.001*). These results are not significantly different from findings in a study in South Africa (25), which showed a prevalence of 18.1% for suicidal risk.

The third perinatal mental disorder observed in mothers was perinatal anxiety, with 10.3% suffering from it. This result is supported by the fact that 10.3% of women had a score of at least 56 on the STAI; 9.3% had a high anxiety score and 1% a very high anxiety score. During the interview, women indicated that these anxiety levels were related to perinatal experiences such as difficult motherhood, fear of recurrent past negative childbearing experiences and fear of the unknown. The study in Rwanda (33), found that the prevalence of perinatal anxiety was 37%, while in ours, it was 10.3%.

These results reveal the extent of the psychological suffering among mothers in the perinatal period consulting at the YGOPH. This highlights the need to set up a psychological care system for mothers at the YGOPH and to carry out similar studies in other health facilities in Cameroon. The justification for planning a response to the needs of women with perinatal mental disorders in hospitals is provided in our study. Furthermore, the finding of this major new information, which is the prevalence (53.6%) of perinatal mental disorders in a tertiary hospital in Cameroon, is linked to a number of risk factors. Data presented in this paper on risk factors of the perinatal mental disorders in Cameroon could be used as reference to support policies and plans geared towards the prevention of these disorders. The presence of risk factors such as a history of depression and/or anxiety before delivery, the absence of practical support, and the presence of at least four risk factors is could serve as an indicator of elevated risk for perinatal mental disorders. Faced with the high prevalence of perinatal mental disorders as a public health problem, it is imperative to address this issue urgently, to alleviate substantial mental illness with problem outcomes for both mother and child (34). Considering that Cameroon is not the only country confronted with this situation, it may be helpful to learn from solutions implemented in other countries and adapt to our context.

In Europe and in some parts of Africa (South Africa for instance), part of the solution is creating positions for psychologists and psychiatrists in perinatal care. In addition, medico-psychological collaboration is highly encouraged. The sensitization and training of mother-child services personnel in all hospitals, including primary health centers, is necessary. The implementation of a prevention policy for pregnant and breastfeeding women based on access to primary care in perinatal mental health, integrated in a graduated and coordinated care system, is needed (35). In addition, the WHO, together with United Nations Fund of Population (UNFPA), in 2008 issued recommendations for women's mental health and child health and development in low- and middle-income countries. The first is to improve the identification of mental health problems during pregnancy and the post-natal period, using screening tools, in order to provide appropriate and timely interventions. The second is to develop the skills of health professionals to conduct a “screening interview” for women's psychological distress, during both the antenatal and the postnatal periods. Third, the development of health services offering appropriate care for the needs of women “in distress” during the perinatal period is recommended (36).

### 4.1. Limitations of the study

Our work has certain limitations. The screening and diagnostic tools (EPDS, STAI, MINI) we used, have not yet been validated for the Cameroonian population. Nevertheless, these tools have already used in other low and middle-income countries like Cameroon (10, 25, 33). Another limitation is that our study involved a small sample and was carried out in a single hospital. This means that the results are difficult to generalize. A further study carried out in non-COVID conditions would also be appropriate. Finally, as there may be some elements of self-selection into taking part in this study, this may also limit generalisabilty to all women and may partially explain such factors as the substantial percentage of this study who were tertiary educated and therefore confident of their literacy skills. Indeed, some mothers refused to participate, telling us that they had no time, and also that they did not have *a priori* mental illness. Which leads us to think that those who refused could also have suffered from mental disorders. But how much, we don't know, hence the slight bias. Thereby, it is clear that many patients at this hospital have unidentified and untreated perinatal mental health problems. A systematic study could clarify this issue.

## 5. Conclusion

Our study confirms our initial hypothesis that the prevalence of common perinatal mental disorders is high at YGOPH, with a predominance of perinatal depression. In contrast to others, we found a significant association between the absence of social support and perinatal mental disorders. Based on current knowledge, this appears to be the first study done on the prevalence of common perinatal mental disorders in a tertiary hospital in Central Africa. Given the harmful consequences of perinatal mental disorders on the health and wellbeing of family members and society, these findings are of a real importance. This highlights the need for institutional screening and management of perinatal mental disorders at the YGOPH and suggests a need to explore the situation in other health facilities in Cameroon and Central Africa.

## Data Availability

The original contributions presented in the study are included in the article/Supplementary Material, further inquiries can be directed to the corresponding author.
